# Short Cationic Peptidomimetic Antimicrobials

**DOI:** 10.3390/antibiotics8020044

**Published:** 2019-04-18

**Authors:** Rajesh Kuppusamy, Mark Willcox, David StC. Black, Naresh Kumar

**Affiliations:** 1 School of Chemistry, University of New South Wales, Sydney, NSW 2052, Australia; r.kuppusamy@ad.unsw.edu.au (R.K.); d.black@unsw.edu.au (D.S.B.); 2 School of Optometry and Vision Science, University of New South Wales, Sydney, NSW 2052, Australia; m.willcox@unsw.edu.au

**Keywords:** peptidomimetics, antibacterials, cationic groups

## Abstract

The rapid growth of antimicrobial resistance against several frontline antibiotics has encouraged scientists worldwide to develop new alternatives with unique mechanisms of action. Antimicrobial peptides (AMPs) have attracted considerable interest due to their rapid killing and broad-spectrum activity. Peptidomimetics overcome some of the obstacles of AMPs such as high cost of synthesis, short half-life in vivo due to their susceptibility to proteolytic degradation, and issues with toxicity. This review will examine the development of short cationic peptidomimetics as antimicrobials.

## 1. Antimicrobial Peptides

Endogenous, usually cationic, peptides are produced by all organisms to defend themselves against potential pathogens [1]. This group of peptides are given the general name of antimicrobial peptides (AMPs). More than 3000 AMPs have been isolated from six kingdoms. 

Naturally occurring AMPs are generally composed of fewer than 50 amino acids mostly in their *L*-configuration. Most AMPs are cationic [2,3] but anionic AMPs also known [4]. AMPs have been classified into groups based on their structures such as β-sheets, α-helices, and extended peptides (http://aps.unmc.edu/AP/main.php; Table 1). The α-helical peptides are the largest group of AMPs and have notable amphipathic characteristics. These possess a tertiary structure with a hinge in the middle [5]. They can be unstructured in aqueous solutions but fold into an α-helix upon binding to lipids in membranes [6]. They often contain the helix stabilizing amino acids alanine, leucine, or lysine [6], as well as the positively charged amino acids lysine and arginine. Cationic β-sheet peptides contain one to five disulfide bridges that help stabilise the peptides into conformationally restricted β-sheets [7]. Their antibacterial activity can be greatly impacted by the number of disulfide bridges in the overall structure [8,9]. Cationic linear peptides have unusual biases in amino acids such as containing large amounts of proline, arginine, or tryptophan [10]. One example for such peptide is histatin which is rich in the amino acid histidine [11,12]. Cationic loop peptides are rich in proline and arginine and cannot form amphipathic structures because of the overexpression of proline residues [13,14]. These peptides adopt a loop formation with one disulfide bridge [15]. There are also cyclic AMPs such as gramicidin S [16].

## 2. Mechanism of Action

AMPs principally target the negatively charged phospholipids in bacterial membranes. The cationic AMPs can outcompete native Mg^2+^ and Ca^2+^ ions bound to lipopolysaccharides in the outer membrane of Gram-negative bacteria, resulting in destabilised regions through which the peptides can pass [17]. Once through the outer membrane, the peptides interact with the cytoplasmic membrane causing depolarization and pore formation [18]. Commonly, these activities are bactericidal [19], but some peptides also interact with internal substances to cause cell death [20,21]. In Gram-positive bacteria, the interaction of cationic AMPs with lipoteichoic acid (LTA) in the cell wall occurs initially [22]. This may lead to activation of autolysins that then go on to cause cell death [23], or the peptides may directly cause death through actions of the cytoplasmic membrane [24]. The outer membrane of eukaryotic cells is generally neutral, which results in reduced affinity for the cationic peptides. Most of the membrane active AMPs are amphipathic, i.e., they contain hydrophobic and hydrophilic groups. After their initial electrostatic interaction with negatively charged membranes, the peptides aggregate at the membrane surface and the hydrophobic part helps insertion into the bacterial membrane [25]. 

**Figure 1:**
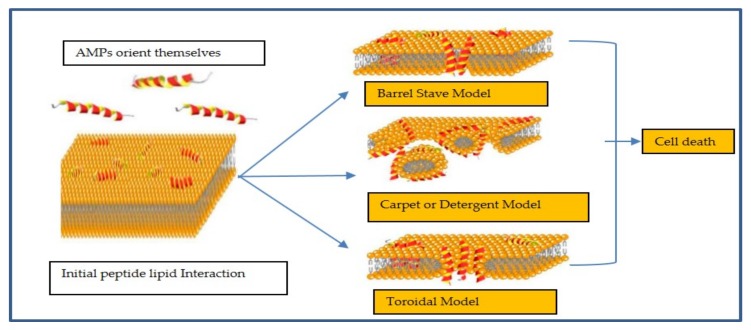
Different models for the action mechanisms of membrane-active AMPs. (modified from Biljana Mojsoska and Havard Jenssen 2015 [34]).

The mechanism of action of membrane active peptides is not fully understood [26]. Their activity and cell selectivity depend on physicochemical parameters of peptides and also on the target membranes [27]. A number of models (Figure 1) have been proposed by which AMPs disrupt membranes [28] (Figure 1). In the barrel-stave-drilling model, AMPs orient themselves perpendicularly to the plane of the membrane bilayer to form pores [29,30]. During this time, AMPs with defined secondary structure undergo orientation in which their hydrophobic groups interacting with membrane lipids and their hydrophilic moieties lining up in the lumen of the pore they create [31]. The AMPs alamethicin, protegrins, and pardaxin act in this barrel-stave fashion [28,32,33]. 

In the carpet model, AMPs accumulate on the bilayer surface [35]. As the concentration of AMPs increases, the membrane is weakened by unfavourable energetics and AMPs are inserted into the membrane in a detergent-like fashion causing the membrane to break into micelles. This mechanism of action does not involve pore formation and, hence, the concentration of AMPs need to be relatively high to cover the bacteria like a carpet [36]. Some examples of AMPs acting by the carpet model are cecropin [37], aurein 1.2 [38], and LL-37 [18]. The toroidal model–wormhole model combines the actions of the barrel-stave and carpet models [39]. The hydrophobic regions of the AMPs associate with the central part of the lipid bilayer with their hydrophilic regions facing the pore [24]. The toroidal model differs from the barrel model in that the peptides are always associated with the lipid head groups even when they are perpendicularly inserted in the lipid bilayer [40]. Some examples of AMPs acting by toroidal model are magainin 2 [41], aurein 2.2 [42], and melittin [40]. 

Since there may be more than one proposed model applicable for the mode of action of AMPs, it is important to understand the sequential steps leading to and then leading from their initial interactions with membranes [41]. Examples of research in this area to more fully understand the mechanism of action is exemplified by research conducted on gramicidin S. Debate has occurred about whether gramicidin S acts by formation of discrete pores or by less specific membrane disruption similar to detergents [43]. Early studies showed that its mechanism of action was due to pore formation [44]. However, more recent studies using cyclic peptide mimics of gramicidin S showed that the mechanism of action involved only with delocalization of peripheral membrane proteins without forming pores [45].

## 3. Mechanism of Resistance

AMPs have been suggested as good alternatives to conventional antibiotics due to their relatively non-specific mechanism of action on bacterial membranes and the rapidity [46] of their antimicrobial action. This has been thought to decrease the chance of resistance development. However, resistance has been demonstrated, and this can be either constitutive, inducible, or acquired.

Inducible resistance to AMPs occurs when the bacteria recognises that AMPs are interacting with it and genes are activated that substitute [47], modify [48], or acylate [49] membrane lipids to reduce their interactions with AMPs. This type of resistance can also include the activation of proteolytic enzymes [50], efflux pumps [51], and modification of intracellular targets [52]. Constitutive resistance to AMPs occurs due to inherent properties of a bacterium that confers resistance and the bacteria express the resistance even in the absence of exposure to peptides. Examples of constitutive resistance include electrostatic shielding of membranes [53], changes in membrane potential during different stages of cell growth [54], and biofilm formation [55]. Acquired resistance to AMPs has been described by horizontal transfer of genes between bacteria [56]. Polymyxin resistance due to the plasmid-mediated *mcr-1* gene was first identified in China in an *Escherichia. coli* strain [57]. Several variants of this gene, *mcr-1-9*, [58] that can confer resistance to polymyxin antibiotics have been identified. However, this gene seems unable to also confer resistance to AMPs such as human cathelicidin LL-37, α-defensin 5 (HD5), or β-defensin 3 (HDB3) [59].

Although bacteria can be or become resistant to AMPs, it is thought that this often requires a high expenditure of energy or substantial changes to the lipid bilayer making resistance development unfavourable [60]. A high level of expression of *mcr-1* decreases cell growth rate and viability, results in degradation of cell membranes and cytoplasmic structures, and so reduces the overall fitness of cell carrying this gene [61]. Also, the relatively low level of resistance to AMPs of bacteria in their natural environments could be due to the combination of the interaction of AMPs with the cell membrane and their ability to act at multiple targets.

## 4. Development of Peptidomimetics

Although AMPs have many favourable qualities, few AMPs have been approved for clinical use, often due to failure to demonstrate efficacy over existing treatments [62,63]. The exception is polymyxins, cationic lipopeptides which are used to treat multi-drug-resistant *Pseudomonas. aeruginosa* infections as a last resort drug [64]. However, polymyxins suffer from significant toxicity problems [65].

The high cost of peptide synthesis and their short half-lives in vivo as a result of their susceptibility to proteolytic enzymes, and issues with toxicity has led to interest in the development of peptidomimetics. Peptidomimetics can be defined as molecules derived from the existing peptides that mimic the biological effect of that peptide. They may have secondary structures and other features to mimic the original peptide and are able to mimic the properties or biological activity of the peptide. The last definition indicates the importance of similar function rather than the similar structure. In this review, most of the peptidomimetic compounds mimic the properties or biological activity of the peptide. 

**Figure 2:**
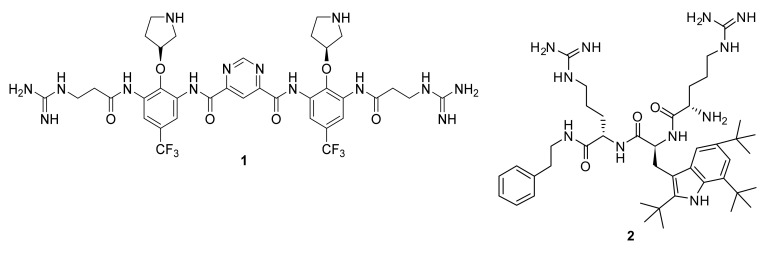
Peptidomimetic compounds in clinical trials.

Short cationic peptidomimetics can overcome some of the challenges currently faced with natural AMPs such as ease of synthesis, increased stability (resistant to the action of proteases/peptidases), and reduced toxicity. Indeed, the peptidomimetics compounds (Figure 2) Brilacidin (**1**) and LTX-109 (**2**) have successfully completed Phase-II clinical trials for skin infection and impetigo [63]. 

Peptidomimetics such as α-peptides, β3-peptides, peptoids, β-peptoids, α/β-peptides, peptide/peptoid hybrids, α-AA peptides, γ-AA peptides, α-oligoacyllysines, and β-oligoacyllysines have been well discussed in a review by Molchanova et. al. [66]. Small-molecule peptidomimetics and glycopeptide antibiotics have been reviewed by Ghosh and Haldar [67,68] including a recent review on bacteriophages as alternatives for conventional antibiotics [69]. Abdel Monaim et al. recently published a review on cyclic AMPs [16] as effective antibacterials. From the key structural features of natural AMPs, peptidomimetic molecules possess an amphiphilic backbone and net positive charge that helps to mimic the biological function of AMPs. Table 2 summarises the research on cationic peptidomimetic antimicrobials by several research groups. In this review, the design of short cationic peptidomimetics as antimicrobials by Australian researchers will be presented according to the theme of this special edition of Antimicrobials “From the Southern Hemisphere: research on resistance, antibiotics and treatments”.

### 4.1. Binaphthyl Peptidomimetics

The group of Bremner, Keller, and Pyne [79] designed a peptidomimetic to mimic the action of vancomycin. Vancomycin disrupts bacterial cell wall synthesis by inhibiting the formation of the strengthening crosslinks in peptidoglycan, which contains terminal *D*-alanine [80]. However, in vancomycin-resistant bacteria such as vancomycin-resistant *enterococci* (VRE), the cross-linking peptide is terminated with *D*-lactate (*L*-Lys-*D*-Ala-*D*-Lac), which results in reduction in binding by vancomycin and, hence, its inability to prevent cross-linking in the bacterial cell wall. 

**Figure 3:**
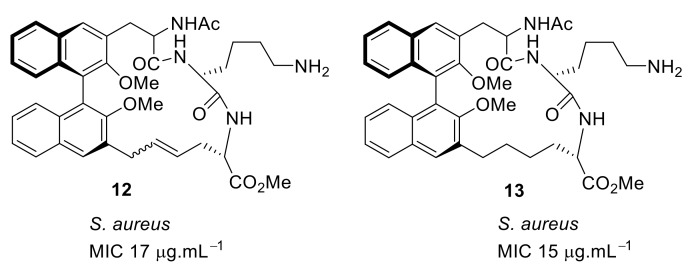
Binaphthyl cyclic peptides.

At first, in order to increase binding interactions, cyclic peptide **12** (Figure 3) based upon a 1,1′-binaphthyl scaffold [79] with tripeptide bridge was investigated. The structure–activity relationship (SAR) was developed by utilizing structural features such as the lysine basic residue for the electrostatic interaction with the carboxy group of terminal *D*-Ala or *D*-Lac, a tripeptide for H-bonding interactions with *D*-Ala and *D*-Lac of VRE and hydrophobic 1,1′-binaphthyl to control the conformation of peptide unit by employing *R* or *S*-enantiomers [79]. The major and minor diastereomer **12** had minimum inhibitory concentrations (MICs) of 17 µg·mL^−1^ and µg·mL^−1^, respectively, against *Staphylococcus. aureus*. However, the hydrogenolysis of a double bond gave compound **13** (Figure 3), which showed MIC against *S. aureus* of 15 µg·mL^−1^. The *E* or the *Z* olefin in **12** was not important for antibacterial activity [79]. The initial binding studies were done using mass spectrometry. The compound **12** indicated a greater affinity to model vancomycin-resistant bacterial cell wall precursor peptide Gly-Ala-Ala-*D*-Ala-*D*-Lac with an unbound to bound ratio of 3.5:1. The cytotoxicity of the compounds were not reported.

**Figure 4:**
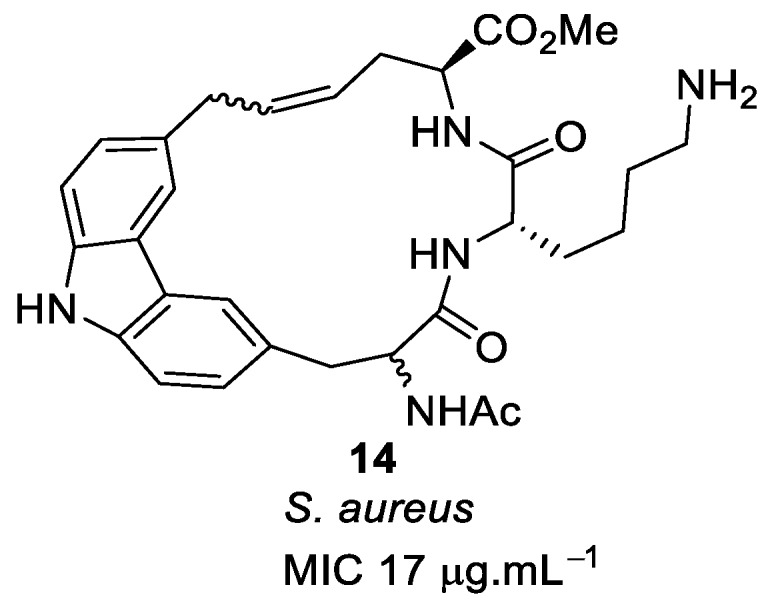
Carbazole peptide.

In another study, a carbazole system was introduced in place of the binaphthyl hydrophobic moiety to improve the antibacterial activity of compound **12**. The carbazole peptide **14** (Figure 4) was synthesised using key ring-closing metathesis to attach different sugar residues through the carbazole NH to mimic the mechanism of action of vancomycin [81]. The carbazole system did not improve the antibacterial activity but gave the same MIC as the binaphthyl system (17 µg·mL^−1^).

**Figure 5:**
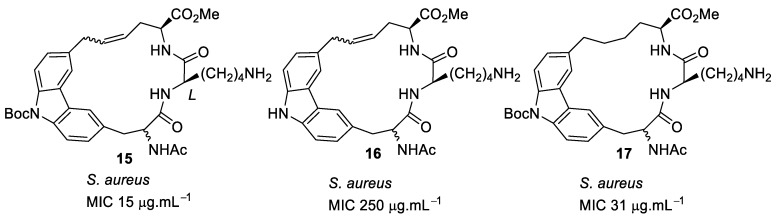
Carbazole-based peptides.

In subsequent structure–activity relationship (SAR) studies, novel carbazole-linked cyclic and acyclic peptides were synthesised [82] with lysine (*D* and *L*) and arginine (*D* and *L*) being used as the basic groups. The corresponding dihydro derivatives were also synthesised by hydrogenolysis of alkene moiety. The SAR study showed that the extra hydrophobicity provided by *N*-Boc compound **15** (Figure 5) increased the antibacterial activity by 16-fold compared to compound **16** (Figure 5). The effect of hydrophobicity was consistent with the *N*-Boc-*L*-lysine dihydro compound **17** (Figure 5). The basic residue lysine showed better antibacterial activity compared to arginine. However, the antibacterial activities were relatively poor (>250 µg·mL^−1^) for the fully protected basic residues, which emphasises the importance of basic groups. The antibacterial activity results suggested that *L*-lysine and a large hydrophobic group were required for maximum antibacterial activity. There was no report about the cytotoxicity and mechanism of action.

**Figure 6:**
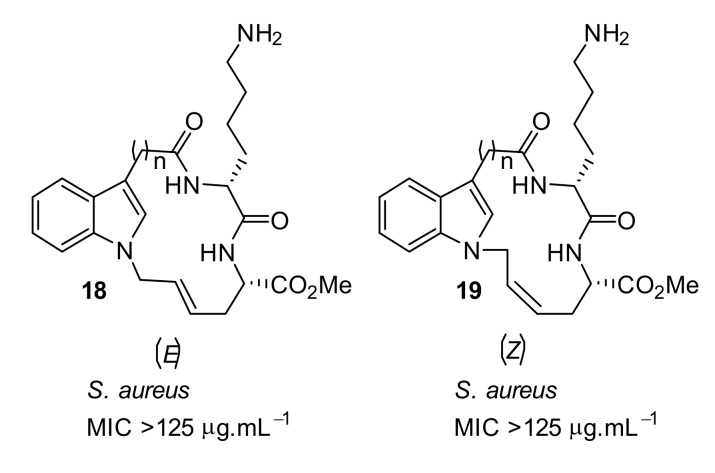
Indole-based peptides.

Another cyclic peptide based on an indole scaffold was explored to study the effect of a smaller rigid scaffold compared to binaphthyl and carbazole scaffold. The indole-based scaffold **18**–**19** (Figure 6) did not show any antibacterial activity, even at 125 µg·mL^−1^. This result suggested that the binaphthyl scaffold might contain a degree of flexibility, thus giving it improved antimicrobial function, however the carbazole and indole scaffolds may be quite rigid and so reduce antimicrobial function.

**Figure 7:**
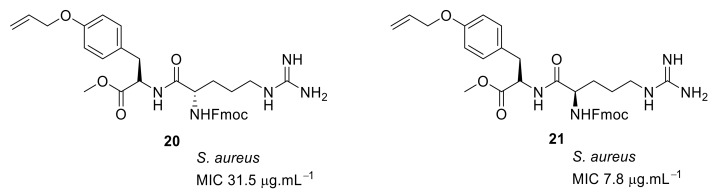
Tyrosine-based peptides.

In order to improve the antibacterial activity, a series of cyclic and acyclic peptide compounds were developed with a simple tyrosine system [83] as the hydrophobic group and the cationic residues from lysine or arginine. In this design, the linear chain showed better antibacterial activity than the corresponding macrocyclic compound. The SAR showed that there was no profound effect on antibacterial activity by changing the lysine cationic residue to arginine. However, the linear chain with an arginine residue in the *D*-configuration of compound **21** (Figure 7) showed good activity against *S. aureus* with an MIC of 7.8 µg·mL^−1^ compared to *L*-configuration of arginine compound **20** with an MIC of 31.5 µg·mL^−1^. The activity of the peptide mimic could be attributed to the presence of the more hydrophobic Fmoc group compared to the tyrosine residue, which is more isosteric to the binaphthyl ring system. This design showed that macrocyclic tyrosine was not necessary for antibacterial activity. The active compounds were not subjected to cytotoxicity or mode of action studies.

**Figure 8:**
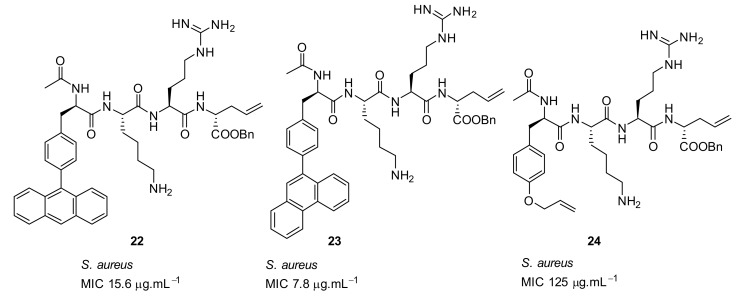
Aryl phenyl alanine and tyrosine-based peptides.

Short cationic peptides using aryl phenylalanine [84] as the hydrophobic group attached with lysine and arginine as cationic groups have been developed. The increase in hydrophobicity with 9-pheneanthrenyl compound **23** (Figure 8) along with two cationic groups increased the antibacterial activity giving an MIC of 8 µg·mL^−1^ against *S. aureus* compared to the less hydrophobic O-allyl tyrosine compound **24** (MIC = 125 µg·mL^−1^; Figure 8). These biological results indicate that two hydrophobic and two cationic groups might be important for antibacterial activity. The active compounds were not subjected to cytotoxicity and mode of action studies.

**Figure 9:**
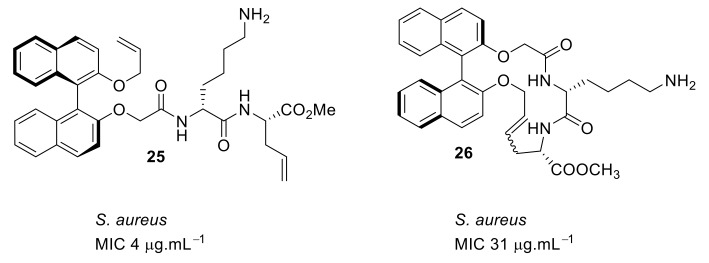
2, 2′-Binaphthyl-based peptides.

Based on the results from the tyrosine system, the binaphthyl hydrophobic cores were reinvestigated. In this design [85], peptides were attached at the 2 and 2′ positions of the binaphthyl system, rather than at the 3 and 3′ positions [79]. The SAR was compared with the cyclic and acyclic counterparts. An acyclic peptide compound **25** with lysine showed excellent antibacterial activity (MIC = 4 µg·mL^−1^) against *S. aureus* ATCC6538 compared to the cyclic cationic peptide **26** (Figure 9). The acyclic cationic peptide **25** (Figure 9) can be easily prepared compared to the more complex cyclic peptide **26**. The active compounds have not been subjected to cytotoxicity and mode of action studies.

**Figure 10:**
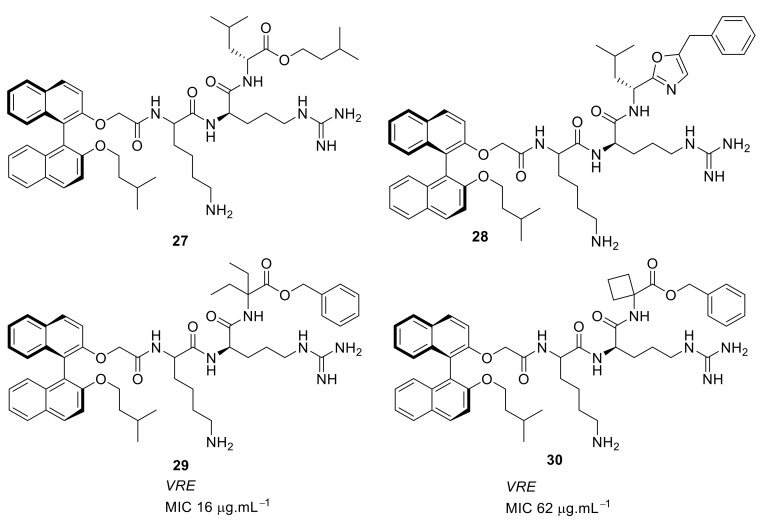
End-chain-modified binaphthyl peptides.

The group of Bremner, Keller, and Pyne examined several cyclic peptide scaffolds based on 1,1′-binaphthyl [79], carbazole [81], indole [86], and tyrosine [83], which all contain cationic amino acid residues to interact with the altered peptidoglycan cell wall of vancomycin-resistant *S. aureus* (VRSE) and vancomycin-resistant *enterococci* (VRE). Using selective modifications on the 1,1′-binaphthyl [87] hydrophobic scaffold, the Lys-Arg-containing acyclic peptide was found to be effective against *S. aureus* and with further modification to the end chain with isopentyl ester and oxazole the effective compounds **27** (MIC = 4 µg·mL^−1^) and **28** (MIC = 4 µg·mL^−1^) were produced (Figure 10; Table 3). Further modification of the end chain hydrophobicity with cyclohexyl substituent [88] in the dicationic tripeptide [87] showed good activity against several gram-positive strains (Table 3). The incorporation of hydrophobic alkyl ring resulted in good activity against *S. aureus* and *S. epidermidis*, but greater variation against *enterococcal* strains. The conformationally less-restricted diethyl-substituted compound **29** showed better activity against vancomycin-resistant *enterococci* (VRE) compared to constrained cycloalkyl compound **30** (Figure 10).

The mode of action of compound **27** was studied using cell-wall model peptide sequences of vancomycin-resistant and vancomycin-sensitive *S. aureus*. Electrospray ionization mass spectrometry (ESI-MS) showed that the vancomycin complexed only with the terminal Ala-sequence, whereas **27** showed 1:1 complex with model peptide sequences together with separate peaks for the individual components. This confirmed that the mode of action of **27** was probably due to inhibition of cell-wall synthesis. The compounds showed bactericidal activity within 2 h at a concentration of 8 µg·mL^−1^, indicating that these compounds may have dual actions. The observed in vitro activity was taken further into in vivo studies. Compound **28** was dosed systematically in an animal model which measured the bacterial growth of methicillin-resistant *S. aureus* (MRSA) in the spleens of infected mice and the number of viable bacteria was reduced after four days of treatment. The control (DMSO vehicle) showed 128 cfu per spleen, while for the compound **28,** only 11 cfu per spleen was observed. The activity of compound **28** was also established topically by using a mouse nasal decolonization model. A single administration of 5 wt% of compound **28** was as active as 2% mupirocin. Furthermore, compound **28** was stable up to 4 h in human plasma. 

**Figure 11:**
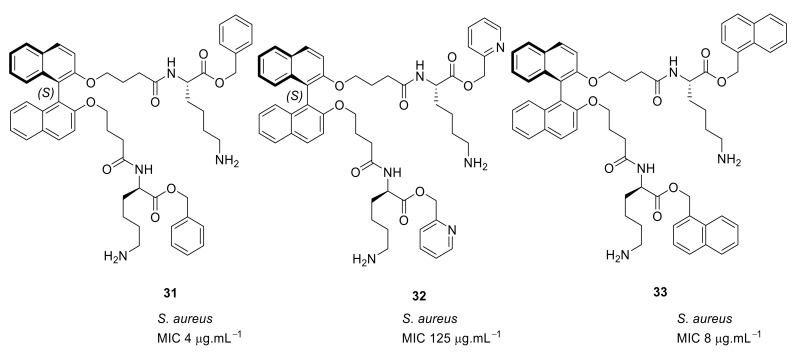
Increased steric bulk binaphthyl peptides.

Binaphthyl [89] hydrophobic scaffolds with cationic amino group substitutions on both 2,2′-oxy positions were further investigated. The SAR was developed by positioning the amino acids sequentially pendant from one of the naphthyl unit hydrophobic scaffold. The different compounds **31–33** (Figure 11) demonstrated that the terminal hydrophobic steric bulk decreases antibacterial activity and extra length of the cationic side chains had no profound effect on antibacterial activity. It was concluded that the compounds with one side chain on the *C*_2_-symmetric scaffold [87] would be sufficient for improved antibacterial activity.

**Figure 12:**
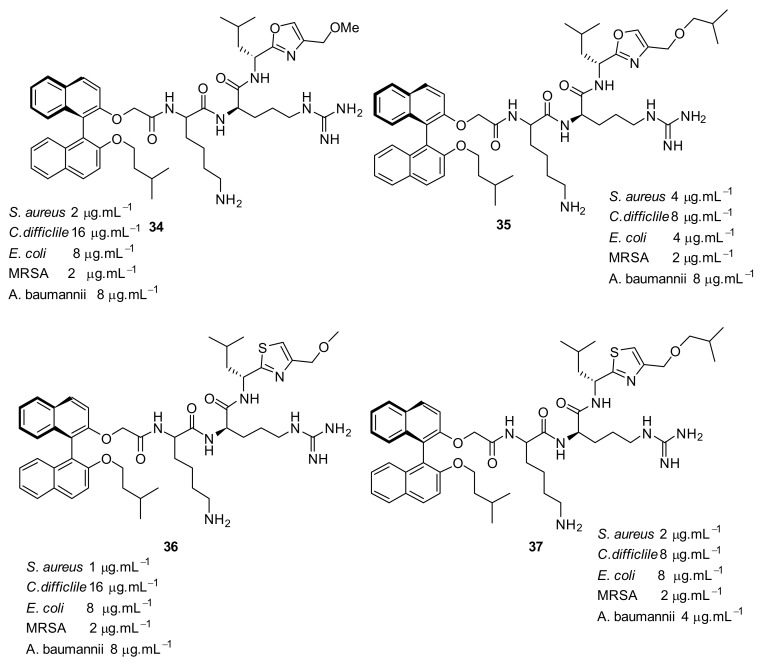
Binaphthyl peptides with oxazole and thiazole substituents.

The second generation of binaphthyl *C*_2_-symmetirc scaffolds were developed with oxazole and thiazole [90] functionalities at the end chain of a dicationic peptide and showed that this produced the most potent binaphthyl peptidomimetics **34**–**37** (Figure 12) against various bacterial strains.

**Figure 13:**
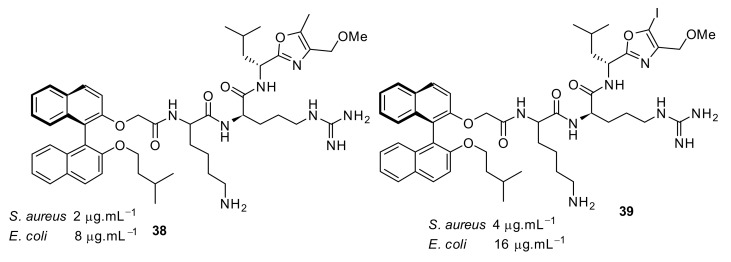
Binaphthyl peptides with oxazole and thiazole substituents.

**Figure 14:**
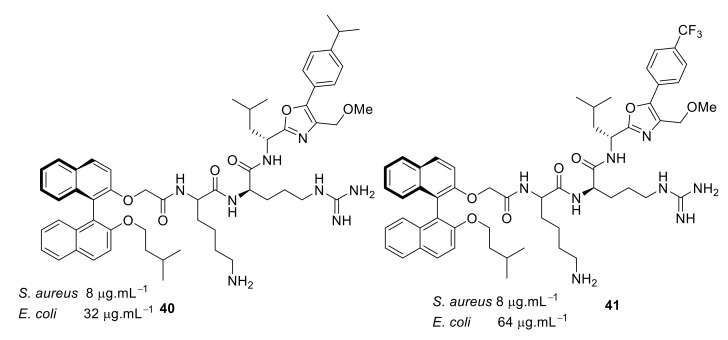
Binaphthyl peptides with increased hydrophobicity in oxazole and thiazole rings.

Increasing the hydrophobicity in the oxazole ring **38**–**41** (Figure 13 and Figure 14) retained antibacterial activity against *S. aureus* but decreased the activity against Gram-negative bacteria. Antibacterial activity decreased up to 8-fold with phenyl substitution. The active compounds **34** and **35** (Figure 12) exhibited <4% hemolysis of sheep erythrocytes at 5.6 µM. However, at approximately 185 µM, they gave >70% hemolysis. Compounds **34** and **35** were not toxic up to their MIC values. The mode of action of these compounds has not been studied.

Table 3 summarises the best antibacterial compounds derived from binaphthyl series by Bremner, Keller, Pyne et al.

### 4.2. Glyoxamides and Carboxamide Peptidomimetics

Kumar et al. have developed peptidomimetics based on glyoxamide and carboxamide scaffolds. Mono- and bis-glyoxamide derivatives and dendrimeric peptide mimics were synthesised by a simple and efficient ring-opening reaction of *N*-acylisatins with a range of amino acids [91,92]. However, the compounds’ antibacterial activity was not reported.

**Figure 15:**
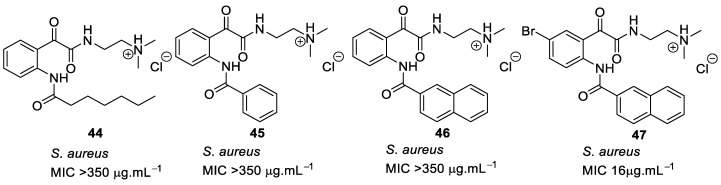
Glyoxamide peptidomimetics with quaternary cationic groups.

The short peptidomimetic compounds [93] based on novel *N*-naphthoyl-phenylglyoxamides were designed (Figure 15). The design investigated the antibacterial effect of cationic tertiary and quaternary salts with different hydrophobic groups. SAR studies demonstrated that the hydrophobicity given by naphthoyl was superior to other hydrophobic groups. A bromo-substituted naphthoyl derivative **47** showed greater antibacterial activity compared to Cl, F, and CH_3_ substituents. Tertiary salts were preferable for antibacterial activity. However, the quaternary ammonium iodide salts (>400 µM) were less toxic to mammalian cells compared to the hydrochloride salts (<40 µM). A tethered bilayer lipid membrane (tBLM) study of the active compound **47** suggested that the antibacterial activity was unlikely to be as a result of membrane rupture.

**Figure 16:**
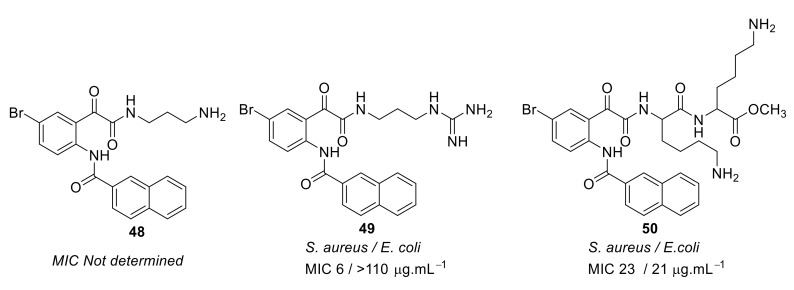
Glyoxamide peptidomimetics with amino and guanidine cationic groups.

To improve the antibacterial activity, the cationic groups were replaced with guanidine [94] or lysine to form dipeptide compounds with increased net charge (Figure 16). The guanidine cationic group compound **49** gave better antibacterial activity (MIC = 6 µg·mL^−1^) compared with the lysine dipeptide compound **50** (MIC = 23 µg·mL^−1^) against *S. aureus*. Similar to previous results [93], bromo-substituted compounds showed better antibacterial activity compared to H, Cl, F, and CH_3_ substituents. The increase in net charge of bromo-substituted compound **50** showed moderate activity against Gram-negative bacteria *E. coli* (MIC = 21 µg·mL^−1^).

**Figure 17:**
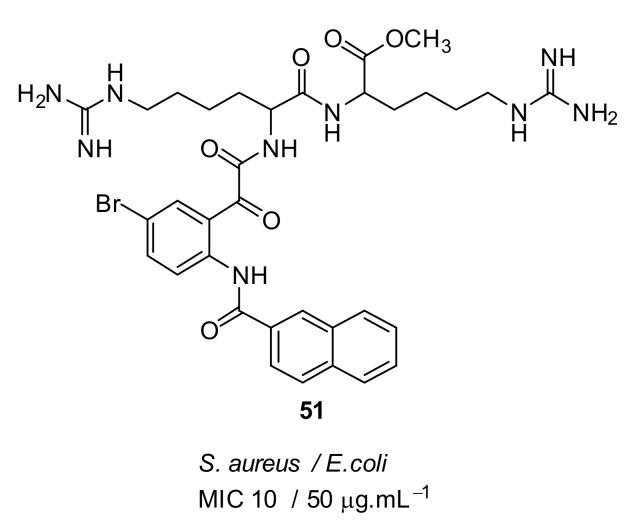
Glyoxamide peptidomimetics with dicationic groups.

The dicationic arginine compound **51** (Figure 17) did not improve antibacterial activity over **50**. Hence, it is notable that simple guanidine with a naphthoyl hydrophobic group is sufficient for antibacterial activity against *S. aureus*. The active compounds **49** and **51** showed low toxicity of 750 µM and 224 µM against human cells. Compounds **49** and **51** were not toxic against mammalian cells. The membrane rupture mechanism of action of compound **49** was investigated using tBLMs. The result showed that the compound did not increase the membrane conduction in bacterial-like negatively charged lipid palmitoyl-oleoyl-phosphatidylglycerol (POPG) and so cell lysis may not occur via the formation of membrane pores. The mechanism of cell death with compound **49** has yet to be established.

**Figure 18:**
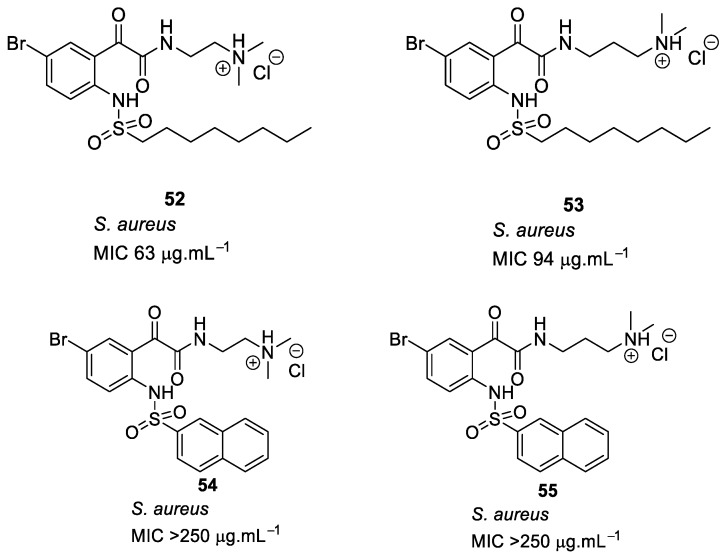
Glyoxamide peptidomimetics with sulfonyl hydrophobic groups.

In a further advancement, the naphthoyl hydrophobic group [93] was replaced with different sulfonyl groups [95], and tertiary ammonium, quaternary ammonium, or guanidinium cationic groups were used to improve the antibacterial activity of glyoxamide compounds (Figure 18). SAR revealed that the bromo-substituted compound **53** with octane sulfonyl hydrophobic group and tertiary ammonium cationic group showed moderate antibacterial activity but was not as effective as the naphthoyl-containing compound **47**. When a naphthoyl sulfonyl hydrophobic group replaced the octane sulfonyl group **54**–**55**, the antibacterial activity was lost.

**Figure 19:**
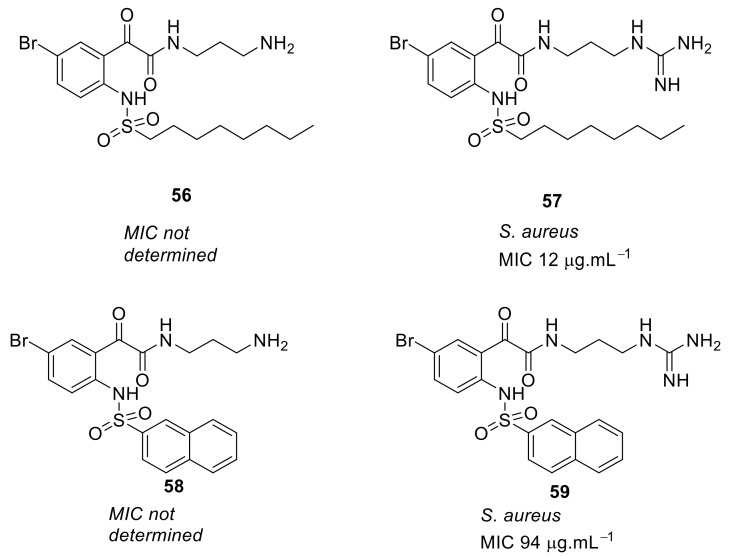
Glyoxamide peptidomimetics with sulfonyl hydrophobic and guanidine cationic groups.

The bromo-substituted octane sulfonyl derivative **57** (Figure 19) with a guanidine cationic group had good antibacterial activity against *S. aureus* (MIC = 12 µg·mL^−1^), and compounds with other cationic groups were not as effective as those with guanidine. The naphthoyl sulfonyl hydrophobic group **59** (Figure 19) improved the antibacterial activity compared to the respective naphthoyl carboxamide hydrophobic-substituted compound **49**. These results suggested that the bromo-substituted scaffold with simple naphthoyl or octane sulfonyl groups and guanidine cationic groups can give improved antimicrobial activity when used in peptidomimetics. The active octane sulfonyl derivative **57** was not toxic (178 µM) against human cells and the mode of action using tBLMs suggests that they may act via membrane disruption.

**Figure 20:**
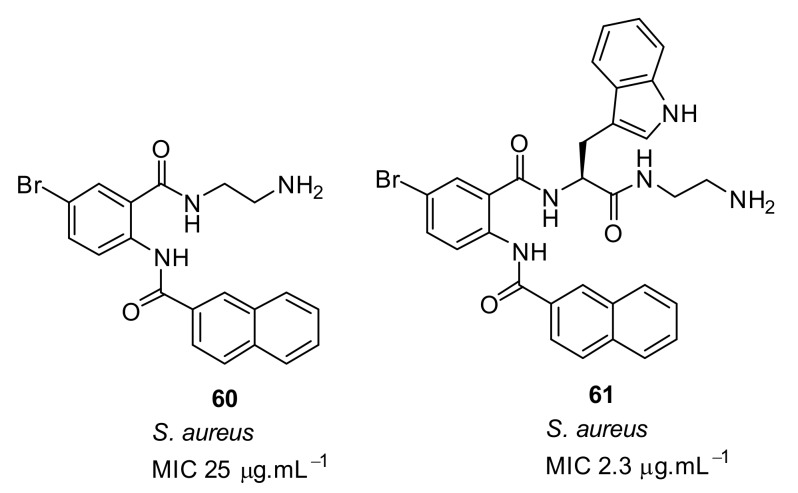
Carboxamide peptidomimetic compounds.

The glyoxamides with naphthoyl hydrophobic groups in combination with different cationic groups showed good antibacterial activity, and hence a study of the effect of glyoxamides and carboxamides on antibacterial activity was conducted (Figure 20). The carboxamide peptidomimetics [96], in which one tryptophan inserted is in compound **60**, dramatically improved the antibacterial activity of compound **61** against gram-positive *S. aureus* (MIC = 2.3 µg·mL^−1^)

**Figure 21:**
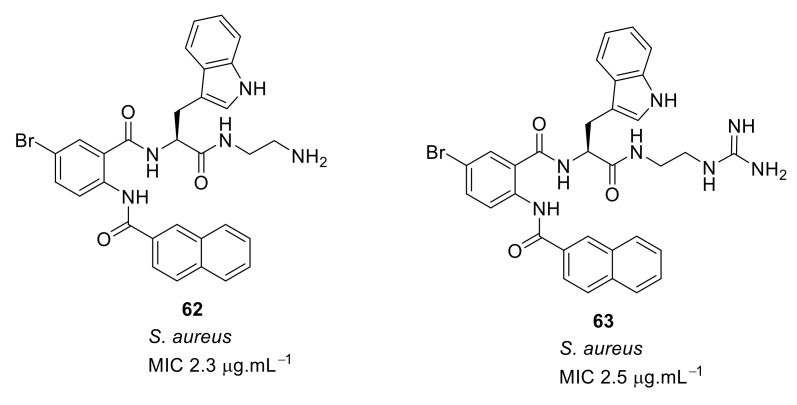
Carboxamide peptidomimetics with different cationic groups.

The SAR was developed using different cationic groups such as amines, guanidines, tertiary, and quaternary ammonium iodides (Figure 21). The halogen-substituted compounds with a naphthoyl hydrophobic group and amine cationic groups showed good antibacterial activities ranging in MICs 4.1–2.3 µg·mL^−1^. Compound **65** gave the least antibacterial activity but compounds with an amino **62** or **63** with a guanidine group showed excellent antibacterial activity against *S. aureus* [96].

**Figure 22:**
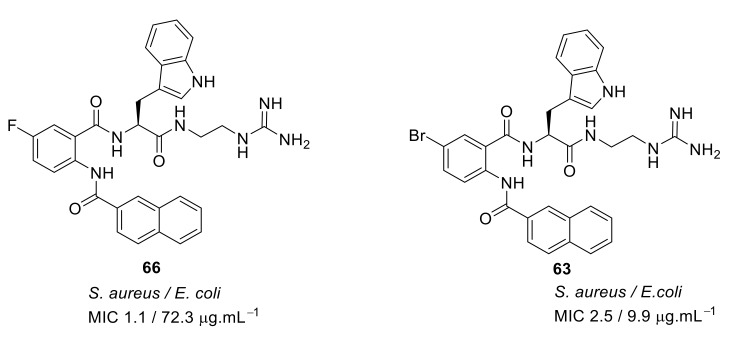
Carboxamide peptidomimetics with guanidine cationic groups.

The fluoro-substituted guanidine derivative 66 (Figure 22) exhibited excellent antibacterial activity against *S. aureus* but was relatively inactive against *E. coli*. However, the bromo-substituted compound **63** (Figure 22) was active against *S. aureus* and *E. coli*. Although the amine cationic groups showed good antibacterial activity against *S. aureus*, they showed toxicity towards mammalian cells. However, the bromo-substituted guanidine **63** was not toxic even at 100 µM concentration [96].

Using bacterial cells loaded with a membrane impermeable dye, addition of the compound **63** caused an increase in fluorescence demonstrating the ability of the compounds to permeate the membrane and cause bacterial cell death [96]. Table 4 summarises the list of best antibacterial compounds derived from glyoxamide and carboxamide peptidomimetics.

### 4.3. Norbornane Peptidomimetics

**Figure 23:**
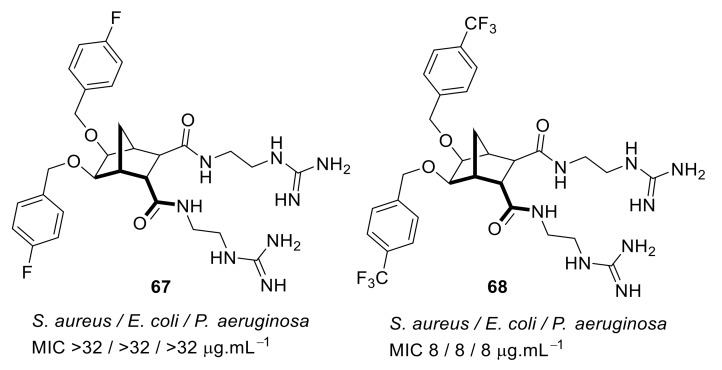
Norbornane peptidomimetic compounds.

Pfeffer et al. developed peptidomimetic compounds using norbornane scaffolds [97,98], and investigated SAR for norbornane bisether diguanidines [99] (Figure 23). Compound **67** with smaller substitutions fluoro on benzyl ethers were not antibacterial, however the compound **68** with larger substituents such as CF3 on benzyl ether showed good antibacterial activity (MIC = 8 µg·mL^−1^). Compounds with higher c log P (i.e., higher hydrophobicity) values showed higher antibacterial activity. However, compound **68** at 100 µM concentration exhibited moderate cytotoxicity with 43% cell survival against human embryonic kidney cells (HEK293) after 24 h. Compound **68** was toxic at a higher concentration than its MIC.

**Figure 24:**
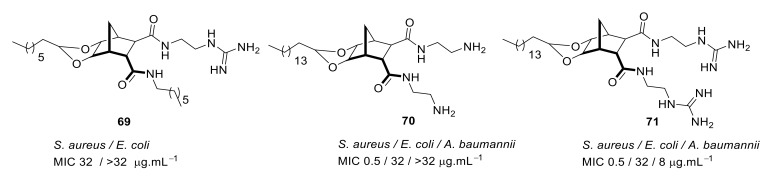
Norbornane peptidomimetics with dicationic groups.

The SAR of norbornanes with different hydrophobic and cationic groups, as well as some neutral anion recognition groups such as thioureas and squaramides, was determined [98]. The dicationic guandinium compound 71 (Figure 24) with a hexadecyl hydrophobic group showed MICs of 0.5–2.0 µg·mL^−1^ against various methicillin-resistant *S. aureus* (MRSA) and vancomycin-intermediate *S. aureus* (VISA) strains. The singularly charged norbornane guanidine compound **69** (Figure 24) showed modest activity compared to the dicationic norbornane **71**, thus demonstrating the importance of net cationic charge. However, the active compound **71** showed toxicity against human embryonic kidney cells (HEK293) at a concentration (6 µg·mL^−1^) approximately equal to its MIC (0.5–32 µg·mL^−1^).

**Figure 25:**
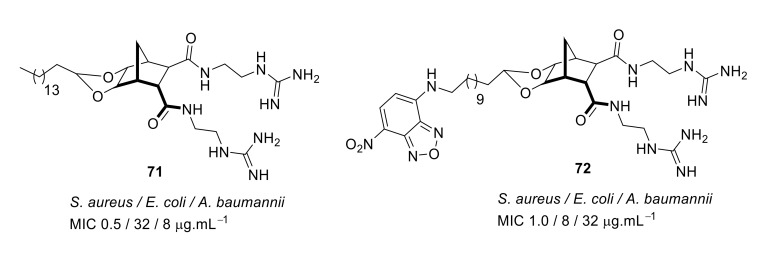
Norbornane peptidomimetic compound attached with fluorophore.

The mechanism of action of the norbornane antibacterial compound was studied by attaching fluorophores [100] (Figure 25). Molecular modelling and fluorescence microscopy studies showed that the compounds **71** and **72** aggregated prior to interacting with cell membranes and fluorescence studies confirmed that these peptidomimetics penetrated into *S. aureus*. Compound **72** was not toxic against mammalian cells.

Table 5 summarises the best antibacterial compounds derived from norbornane cationic peptidomimetics.

### 4.4. Biaryl (Biphenyl) Peptidomimetics

**Figure 26:**
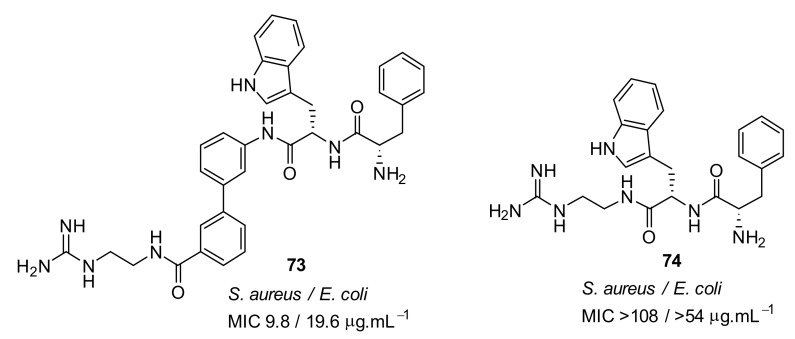
Biphenyl peptidomimetic compound.

Kuppusamy et. al utilised the hydrophobic biphenyl backbone [101] for their peptidomimetic compounds (Figure 26). A SAR was developed by tuning the hydrophobic (Trp/Phe) and cationic groups (Lys/Arg). Upon removing the biphenyl from the active compound **73**, the antibacterial activity of the compound **74** was lost. Thus, the amphipathic nature of the compound was shown to be very important for antibacterial activity [101].

**Figure 27:**
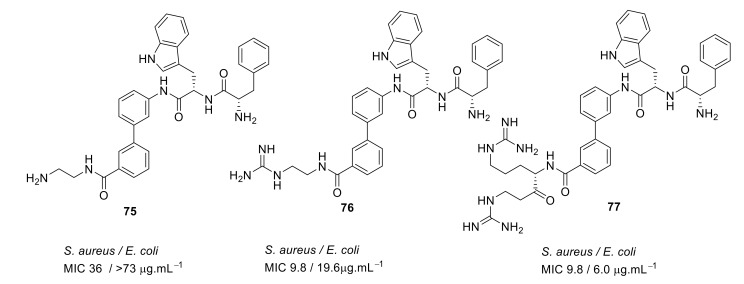
Biphenyl peptidomimetic compounds with different cationic groups.

A simple diaminoethane substituent **75** (Figure 27) was enough to mimic the lysine cationic residue and the guanidine substituent of **76** (Figure 27) was able to mimic an arginine residue. Increasing the net charge of compound **77** increased the antibacterial activity against gram-negative bacteria. These active compounds **75**–**77** (Figure 27) were not toxic to mammalian cells even at a concentration of 400 µM [101].

**Figure 28:**
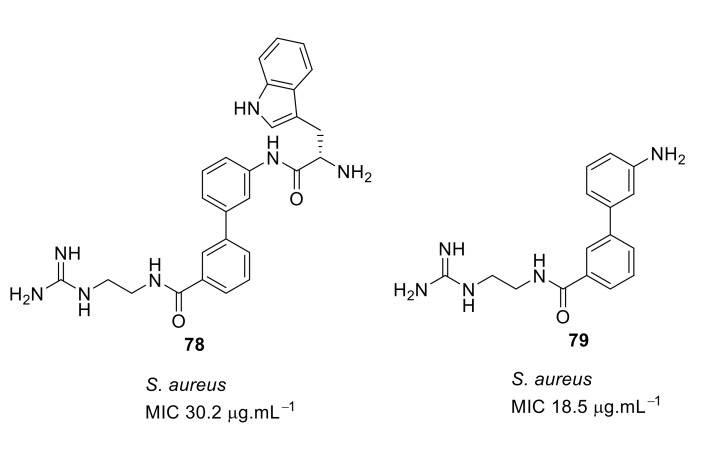
Biphenyl peptidomimetic compound without tryptophan hydrophobic group.

The compound’s ability to interact with lipid bilayers was tested using tBLMs and AC electrical impedance spectroscopy [102,103]. The change in conductance of biphenyl compounds **77**–**79** (Figure 28) with negatively charged membranes demonstrated that the effectiveness of these compounds was not so much related to their overall cationic charge but rather to their ability to insert into a lipid bilayer [102,103]. This means that tryptophan played a crucial role in membrane permeability. Using bacterial cells loaded with a membrane impermeable dye, addition of the biphenyl compound **77** caused an increase in fluorescence demonstrating the ability of the compounds to permeate the membrane and cause bacterial cell death.

Tague et al. have developed biphenyl peptidomimetic compounds [104] based on their most active binaphthyl peptidomimetic compound **38**, but this compound showed toxicity against mammalian cell. Cytotoxicity is the major problem for the further development of some peptidomimetics into antimicrobial drugs. The SAR was developed by comparing the pharmacophores binaphthyl and biphenyl to improve the cytotoxicity [104].

**Figure 29:**
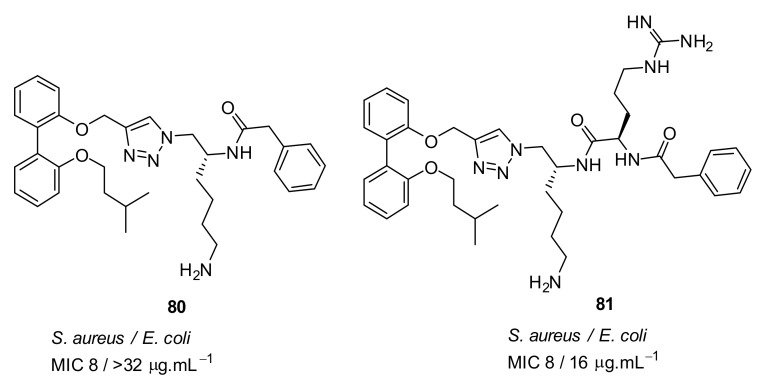
Biphenyl peptidomimetic compound without tryptophan hydrophobic group.

The antibacterial activity against gram-negative bacteria of biphenyl pharmacophore compound **81** (Figure 29) with net charge of +2 was improved compared to the compound **80** with a net charge of +1 [104].

**Figure 30:**
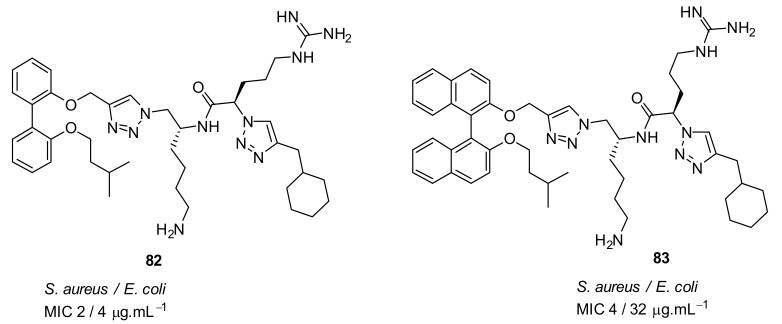
Biphenyl peptidomimetic compound with two triazole bioisostere for amide.

The incorporation of a second bioisostere triazole in dicationic derivatives of biphenyl **82** gave an 8-fold increase in activity against *E. coli* compared to binaphthyl derivative **83** [105] (Figure 30).

**Figure 31:**
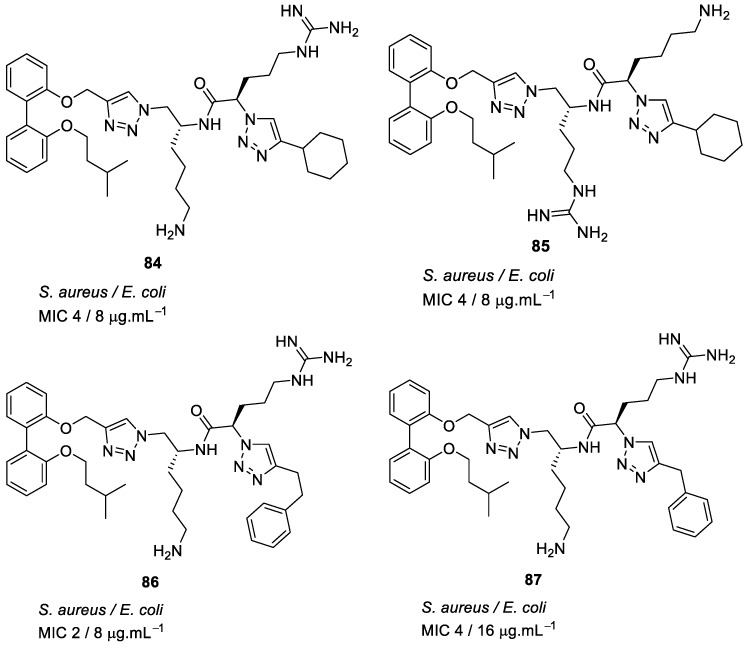
Biphenyl peptidomimetic compounds with different hydrophobic terminal residues.

The antibacterial potency varied with different hydrophobic terminal residues. Flexible hydrophobic groups such as phenethyl **86** and cyclohexyl methyl **82** at the terminus were slightly more potent against *S. aureus* and *E. coli* compared to compounds with cyclohexyl **84** and benzyl termini **87** [104] (Figure 31).

Changing the dicationic group Arg-Lys to Lys-Arg of compound **85** to **84** (Figure 31) did not impact the antibacterial efficacy. The cytotoxicity of biphenyl derivative **82** (42.4%), measured as a percentage of hemolysis, was less compared to the binaphthyl compound **83** (88.8%) which was haemolytic at 66 and 58 µM. Compound **82** was not toxic to mammalian cells at concentrations greater than its MIC value (2–4 µg·mL^−1^).

Using bacterial cells loaded with non-permeable dye (propidium iodide), addition of compound **82** caused a 600% increase in fluorescence confirming that the membrane has been disturbed, and hence the dye enters the cytoplasm and stains the DNA which causes the increase in fluorescence. 

Table 6 summarises the best antibacterial compounds derived from biaryl cationic peptidomimetics.

### 4.5. Short Cationic Lipopeptides

**Figure 32:**
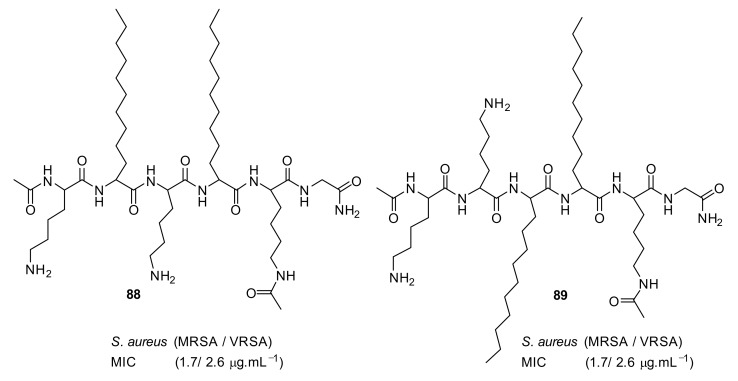
Lipopeptide compounds with alternate and distinct lysine cationic groups.

Short cationic peptidomimetic compounds (Figure 32) based on lipopeptides have been developed by Azmi et al. [106] The lipophilicity and the cationic charge of compounds is driven by C_12_ lipoamino acids (*D, L*-dodecanoic acid) and lysine residues. The SAR was studied by positioning the lysine residues in lipopeptides.

The lysine and C_12_-lipoamino acid (C_12_-LAA) arranged alternatively in compound 88 and the lysine and C_12_-LAA residues grouped in distinct segments in compound **89** almost showed similar antibacterial activity against several *S. aureus* strains [106].

**Figure 33:**
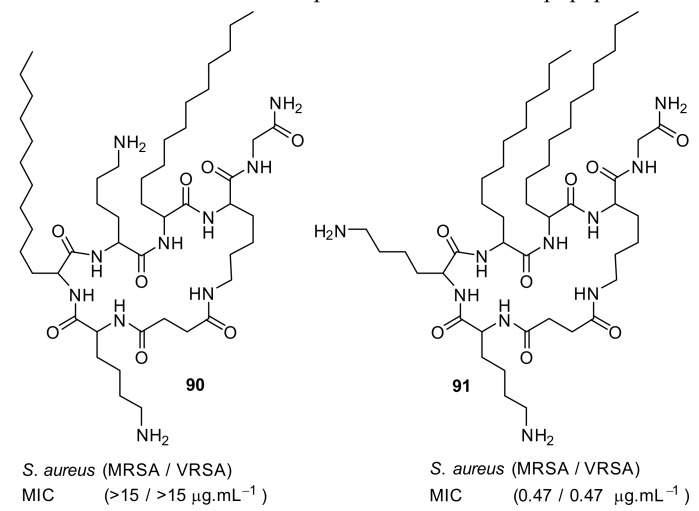
Cyclic lipopeptide compounds with alternate and distinct lysine cationic groups.

The cyclic derivative **90** (Figure 33) with lysine and C_12_-LAA arranged alternatively lost antibacterial activity compared to the distinct segment pattern of the cyclic derivative **91**. The lipopeptides 88 and 91 showed negligible hemolytic activity at 100 µM concentration against mammalian cells. [106] These compounds can self-assemble and form nanoparticles in aqueous environments. Due to this, the cationic charge density is increased, and this may lead to greater bacterial cell selectivity over mammalian cells [106,107]. The mechanism of action was not reported. Table 7 summarises the best antibacterial compounds derived from lipopeptide mimics.

## 5. Conclusions

The increasing emergence of antibiotic resistance has led to the search for alternatives. Short cationic peptidomimetic compounds can mimic the mechanism of action of AMPs and it is possible to quickly synthesise large quantities of them. The examples shown throughout this review illustrate how different scaffolds may be utilised for development of antibacterials. Generally, all these examples contain the concept of specific hydrophobic and cationic groups. The backbone (hydrophobic) has often been shown to be important for the antibacterial activity; for example the removal of biphenyl group led to the loss of antibacterial activity. Structural diversity has been created by modifying the backbone utilizing bioisosteres and heterocycles. Substitution in the backbone also affects the antibacterial activity. For example, bromo-substituted glyoxamides and carboxamides enhanced the antibacterial activity over other halides. The cationic groups lysine and arginine can be replaced with simple amine and guanidine to retain the antibacterial activity. The amphiphilicity of compounds is important and the overall charge and hydrophobicity must be balanced to achieve optimal activity.

There is evidence that AMPs act by multiple mechanisms, but not all studies have undertaken mechanistic studies of peptidomimetic compounds. AMPs can show synergy with conventional antibiotics, but synergistism of short cationic peptidomimetics remains largely unexplored. Considering the success of brilacidin and LTX-109 in Phase-II clinical trials, this class of compounds with different backbones, unnatural amino acids, can be exploited as potential antibacterials.

## Figures and Tables

**Table 1:** Structural Statistics of 3058 antimicrobial peptides (AMPs) in the Antimicrobial Peptide Database (APD) database.

Structural Class	Number of AMPs	Percentage
α-helix	465	15.2%
β-structure	82	2.68%
Mix of α-helix and β-sheet	106	3.46%
Extended	100	3.27%
Disulfide bridge	493	16.12%
Unknown	1789	58.5%

**Table 2:** Peptidomimetic compounds produced using different scaffolds and their minimum inhibitory concentrations (MIC) to various bacteria.

Structure	MIC µg·mL^−1^	Ref
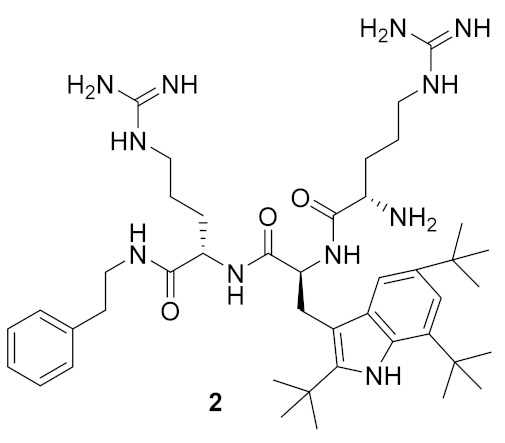	*S. aureus* (3) *E. coli* (8) *P. aeruginosa* (5) Methicillin-resistant *S. aureus* (3) Methicillin-resistant *S. epidermidis* (1) glycopeptide-intermediate *S. aureus* (3)	Isaksson et al. [70]
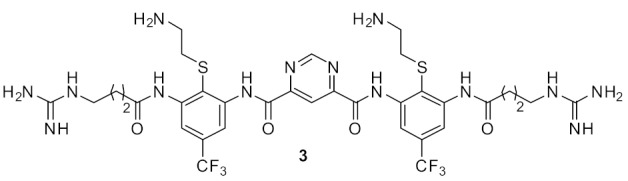	*S. aureus* (0.045) *E. coli* (0.4) *K. pneumoniae* (2) *P. aeruginosa* (2)	Choi et al. [71]
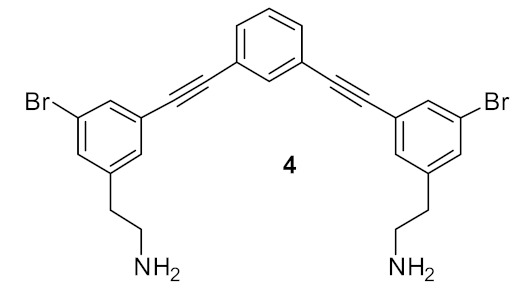	*S. aureus* (0.5) *E. coli* (1)	Isaksson et al. [70]
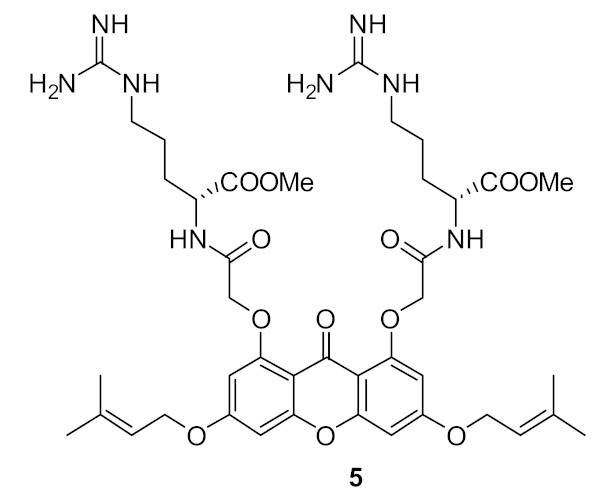	*S. aureus* (0.5) *Bacillus cereus* (2) Methicillin-resistant *S. aureus* (2)	Koh et al. [72]
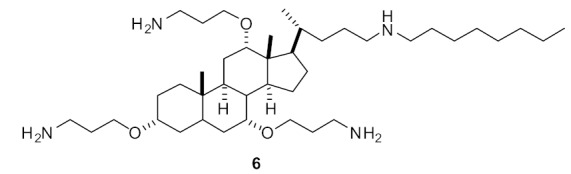	*S. aureus* (0.5) *E. coli* (2)	Bucki et al. [73]
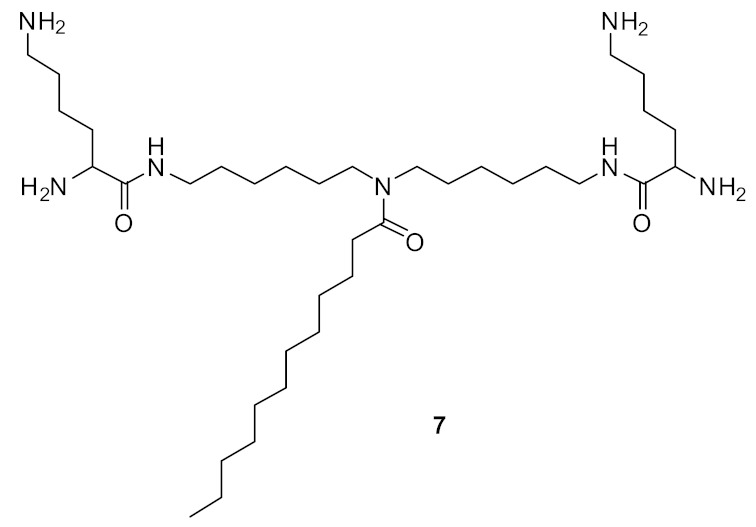	*S. aureus* (2) *E. coli* (3) *P. aeruginosa* (3) Methicillin-resistant *S. aureus* (3)	Konai et al. [74]
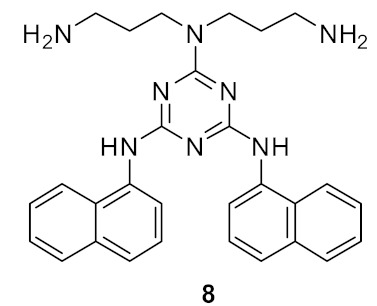	*S. aureus* (3) *E. coli* (5) *P. aeruginosa* (5)	Gunasekaran et al. [75]
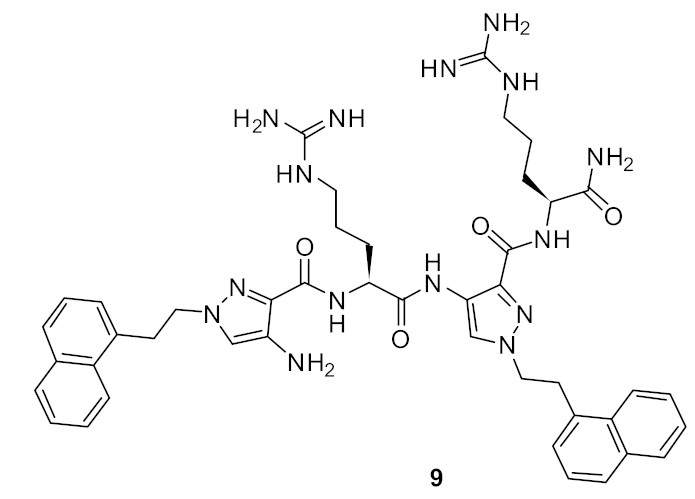	*S. aureus* (4) *E. coli* (32) *P. aeruginosa* (8) Methicillin-resistant *S. aureus* (8) Vancomycin-resistant *E. faecalis* (8)	Ahn et al. [76]
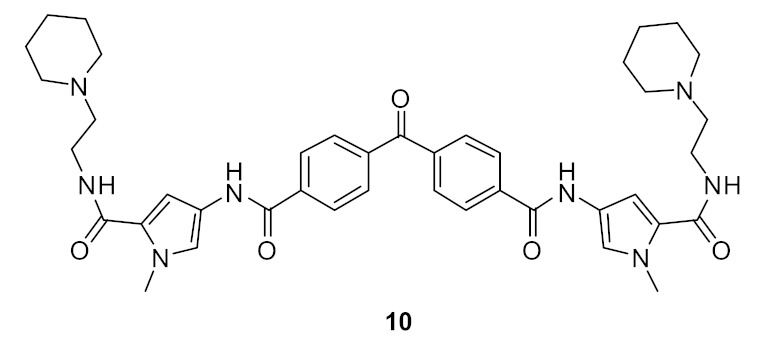	Methicillin-resistant *S. aureus* (1) Vancomycin-resistant *S. aureus* (0.5) *S. aureus* (4)	Vooturi et al. [77]
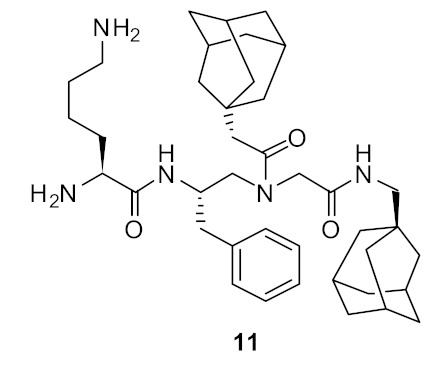	Methicillin-resistant *S. aureus* (2) Vancomycin-resistant *enterococci* (6) Methicillin-resistant *S. epidermidis* (3) *E. coli* (3) *P. aeruginosa* (6)	Teng et al. [78]

**Table 3:** In vitro antibacterial activities of binaphthyl series compounds against Gram-positive and Gram-negative isolates.

Structure	MIC µg·mL^−1^	Ref
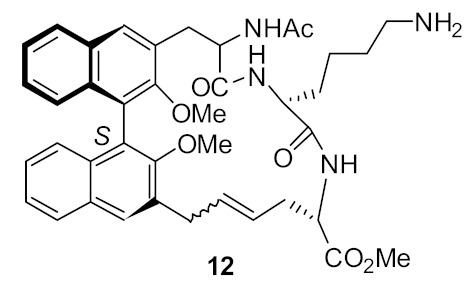	*S. aureus* (17)	Bremner et al. [79]
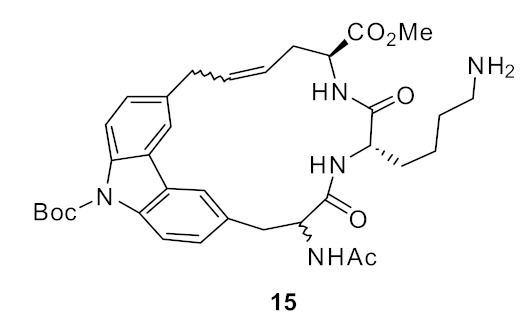	*S. aureus* (15)	Bremner et al. [81]
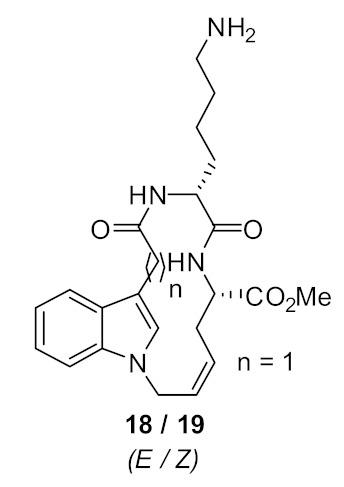	*S. aureus* (>125)	Au et al. [86]
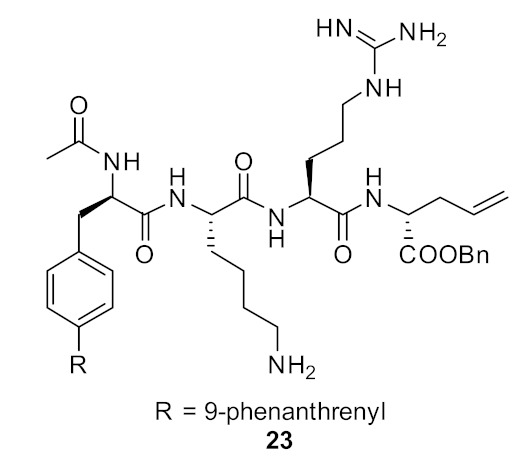	*S. aureus* (8)	Boyle et al. [84]
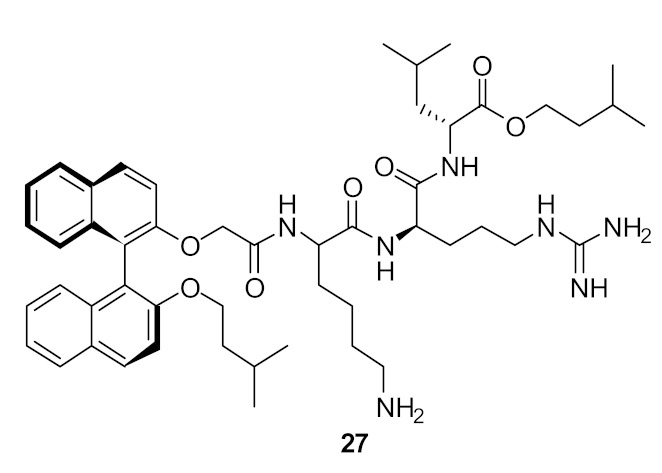	Methicillin-resistant *S. aureus* (2–4) Methicillin-sensitive *S. aureus* (4) Vancomycin-resistant *S. aureus* (2) *S. epidermidis* (4) Vancomyin-resistant *enterococci* (4) A. *baumannii* (4) *E. coli* (16)	Bremner et al. [87]
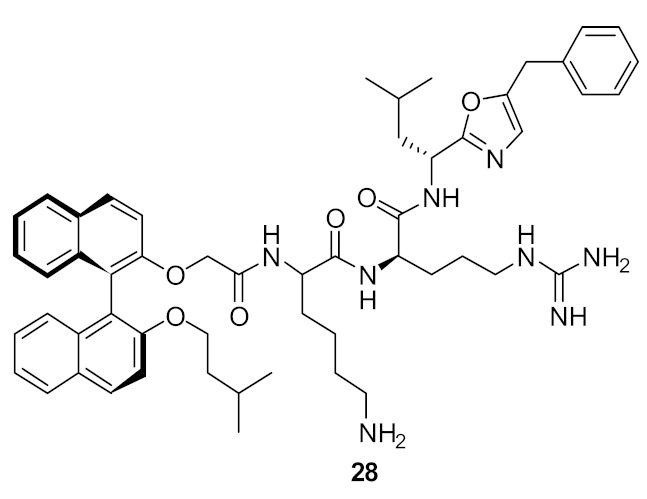	Methicillin-resistant *S. aureus* (4) Methicillin-sensitive *S. aureus* (4) Vancomycin-resistant *S. aureus* (4) *S. epidermidis* (4) Vancomyin-resistant *enterococci* (4) A. *baumannii* (8) *E. coli* (32)	Bremner et al. [87]
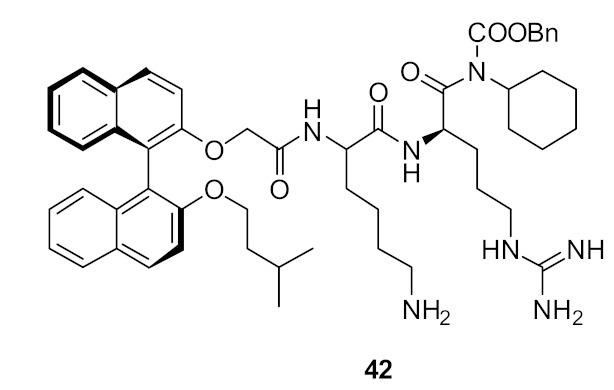	Methicillin-sensitive *S. aureus* (4) Methicillin-resistant *S. aureus* (4) Vancomycin-intermediate *S. aureus* (4) *S. epidermidis* (2)	Bremner et al. [88]
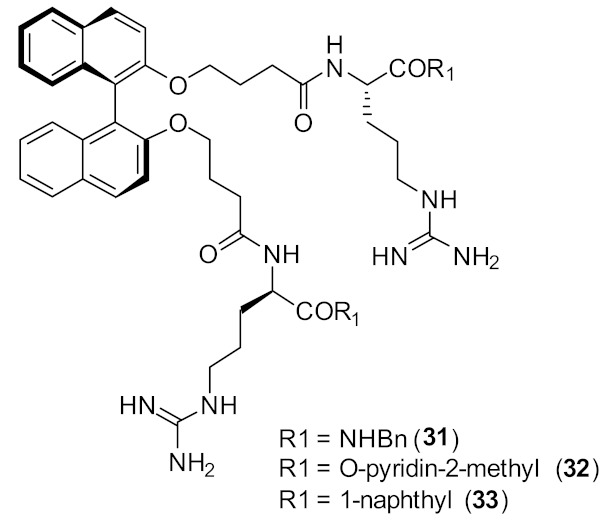	*S. aureus* (4/8) (Bn (**31**)/Nap (**33**)) Vancomycin-resistant *S. aureus* (83/>125) (Bn (**31**)/Nap (**33**))	Robertson et al. [89]
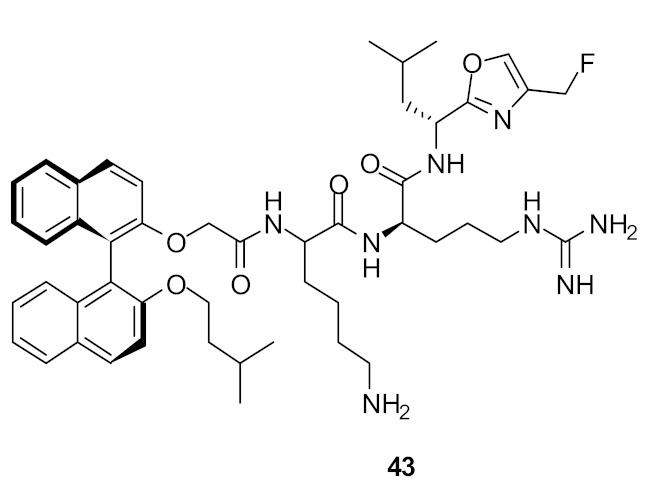	*S. aureus* (1) MRSA (2) *E. faecalis* (2) *S. pneumoniae* (2) *E. coli* (4) *A. baumannii* (4) *C. difficile* (8)	Wales et al. [90]

**Table 4:** Antibacterial activity of glyoxamide and carboxamide compounds against Gram-positive and Gram-negative isolates.

Structure	MIC µg·mL^−1^	Ref
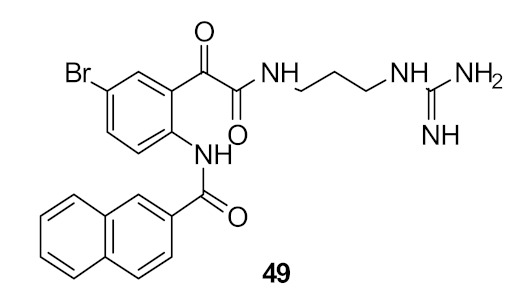	*S. aureus* (6) *E. coli* (>110)	Nizalapur et al. [94]
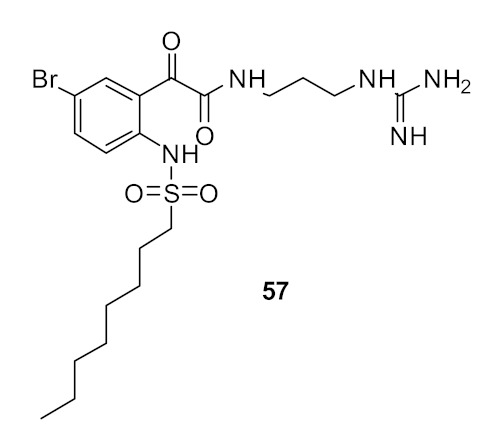	*S. aureus* (12)	Yu et al. [95]
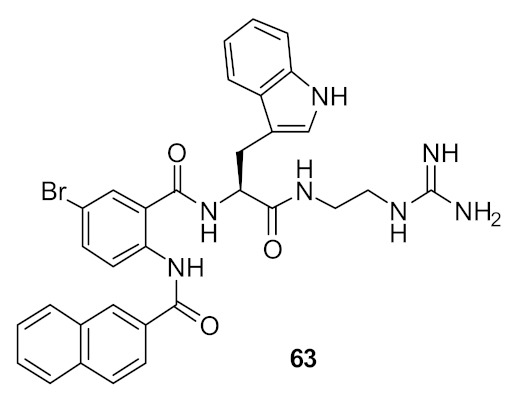	*S. aureus* (3) *E. coli* (10)	Kuppusamy et al. [96]

**Table 5:** Antibacterial activity of norbornane cationic compounds against Gram-positive and Gram-negative isolates.

Structure	MIC µg mL^−1^	Ref
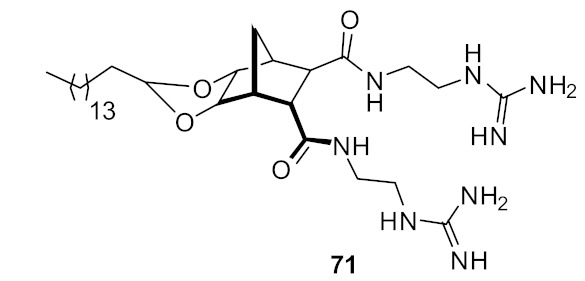	*S. aureus* (0.5) *E. coli* (32)	Henderson et al. Hickey et al. [97,98]
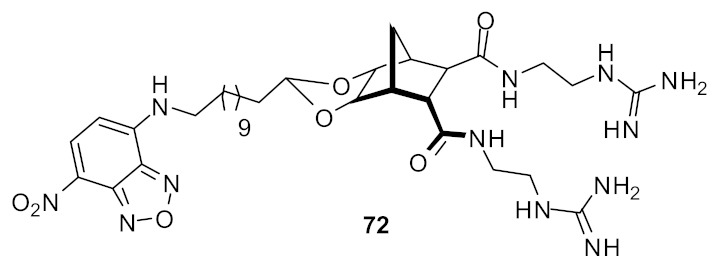	*S. aureus* (1) *E. coli* (8) *S. pneumoniae* (1)	Hickey et al. [100]

**Table 6:** Antibacterial activity of biaryl cationic compounds against Gram-positive and Gram-negative isolates.

Structure	MIC µg mL^−1^	Ref
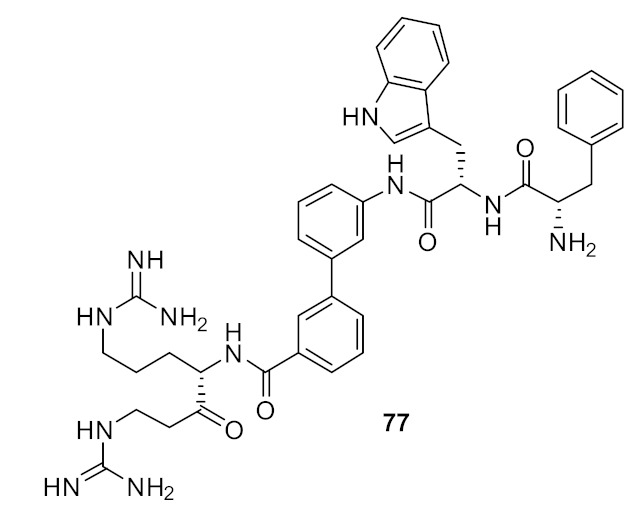	*S. aureus* (10) *E. coli* (6)	Kuppusamy et al. [101]
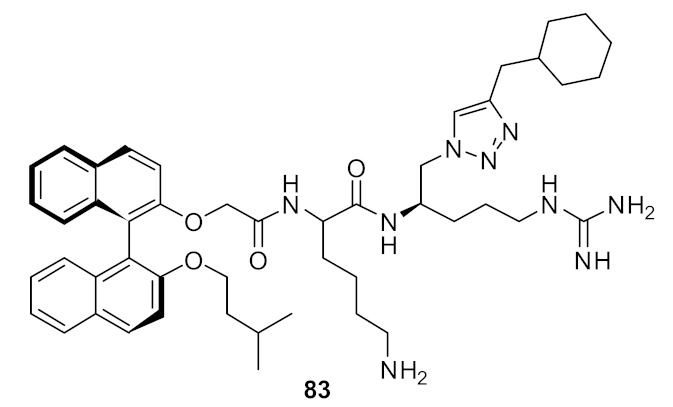	*S. aureus* (4) *E. coli* (32) Vancomyin-resistant *enterococci* (4) *C. difficile* (4) *A. baumannii* (4)	Wales et al. [105]
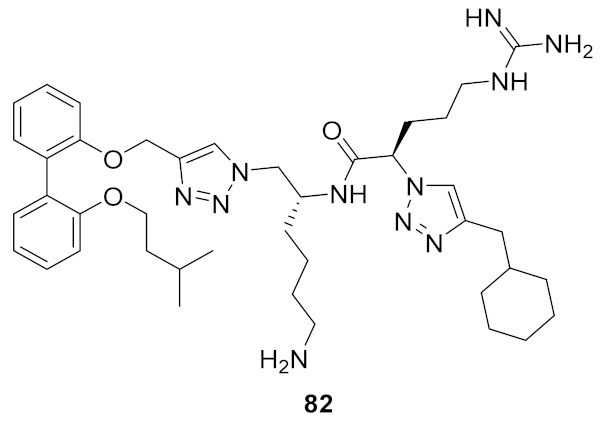	MRSA (2) *E. coli* (4) *C. difficile* (4) *A. baumannii* (8)	Tague et al. [104]

**Table 7:** Antibacterial activity of lipopeptides against Gram-positive and Gram-negative isolates.

Structure	MIC µg·mL^−1^	Ref
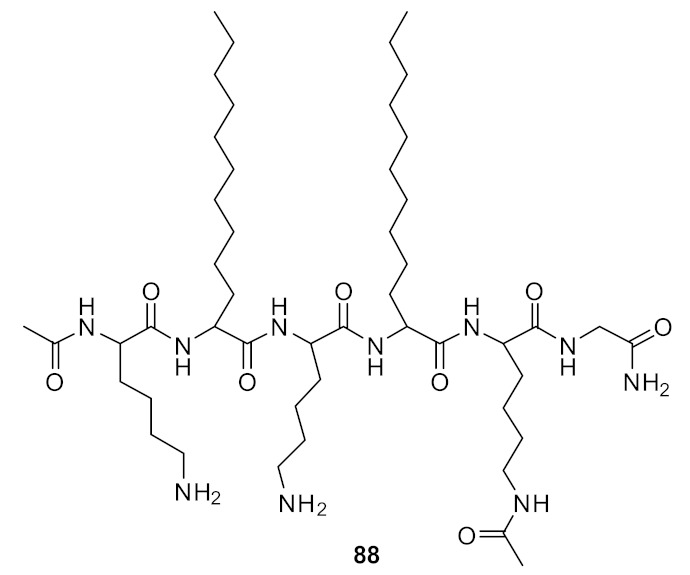	methicillin-resistant *S. aureus* (1.7) Glycopeptide-intermediate *S. aureus* (7.5) Vancomycin-resistant *S. aureus* (2.6) *S. pneumoniae* (5.6)	Azmi et al. [106]
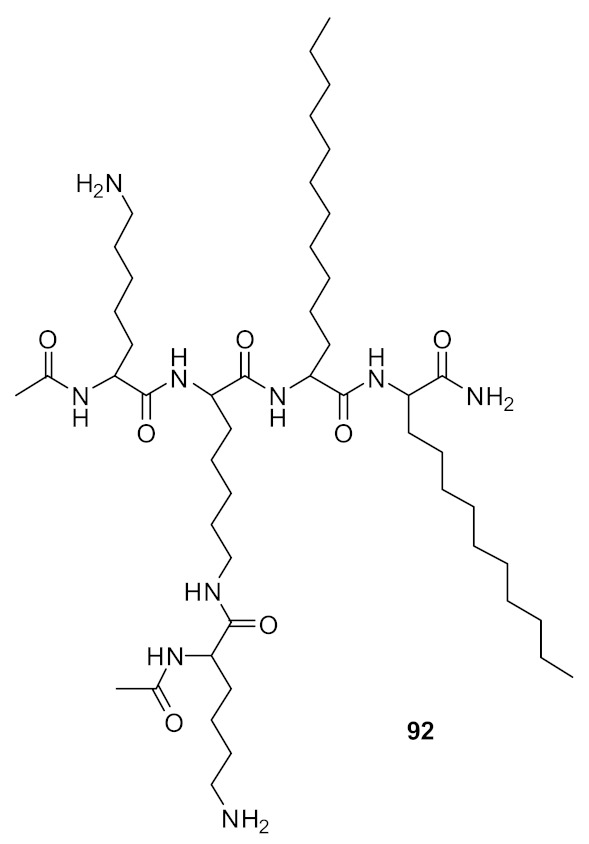	methicillin-resistant *S. aureus* (0.47) Glycopeptide-intermediate *S. aureus* (7.5) Vancomycin-resistant *S. aureus* (0.47) S. pneumoniae (2.8)	Azmi et al. [106]
